# A Novel Ge-Doping Approach for Grain Growth and Recombination Suppression in Buffer-Free CIGSe Solar Cells

**DOI:** 10.3390/ma19030499

**Published:** 2026-01-27

**Authors:** Mengyao Jia, Daming Zhuang, Ming Zhao, Zhihao Wu, Junsu Han, Yuan He, Jihui Zhou, Maria Baranova, Wei Lu, Qianming Gong

**Affiliations:** 1 School of Materials Science and Engineering, Tsinghua University, Beijing 100084, China; jiamy21@mails.tsinghua.edu.cn (M.J.);; 2 Key Laboratory for Advanced Materials Processing Technology of Ministry of Education, Beijing 100084, China; 3 State Key Laboratory of New Ceramics and Fine Processing, Tsinghua University, Beijing 100084, China

**Keywords:** CIGSe, Ge-doping, crystallinity, carrier recombination, buffer-free

## Abstract

**Highlights:**

**What are the main findings?**

- Ge doping promotes grain growth in CIGSe absorbers.
- Ge does not introduce notable impurity phases as a result of its loss via volatile Ge-Se compounds.
- Ge-doping enhances band bending at grain boundaries and suppresses carrier recombination.
- Ge-doping leads to the device performance enhancement of CIGSe buffer-free solar cells.

**What are the implications of the main findings?**

- Provides a novel approach to enhancing the crystallinity of CIGSe thin films.
- Demonstrates the effects of grain boundary passivation on suppressing recombination.
- Offers a method for improving the performance of CIGSe cells with a simplified structure.

**Abstract:**

Ge-doped CIGSe absorbers were fabricated using a two-step process of depositing sputtered stacked Ge-doped CIGSe precursors and selenization annealing. The effects of Ge doping on the crystallinity as well as defects of CIGSe absorbers and the performance of CIGSe buffer-free solar cells were investigated. The results show that Ge doping significantly promotes the grain growth of CIGSe absorbers. Due to Ge loss via volatilization during selenization annealing, Ge residue is undetectable in Ge-doped absorbers. Ge doping offers an effective approach to improve CIGSe crystallinity without introducing notable impurity phases or Ge-related defects. However, Ge doping also induces Se loss, and excessive Se vacancy defects adversely affect the performance of the absorber. In addition, Ge doping increases the contact potential difference at CIGSe grain boundaries and is beneficial for reducing carrier recombination at these sites. Analysis of recombination rates in Ge-doped CIGSe buffer-free solar cells reveals that the combined effects of enhanced crystallinity and optimized electrical properties at grain boundaries effectively suppress the recombination in the space charge region, at the interface, and in the quasi-neutral region, leading to improved device performance.

## 1. Introduction

Driven by the increasing global energy demand and the need to mitigate carbon emissions, solar energy has become one of the most important renewable and clean energy sources. Following extensive research and development, photovoltaic technologies, including crystalline silicon [1], CdTe [2,3], CIGSe [4,5], GaAs (III-V-based) [6,7], and perovskites [8,9], have achieved remarkable advancements and efficiency breakthroughs. Among these materials, Cu(In,Ga)Se_2_ (CIGSe) thin-film solar cells are considered one of the most promising photovoltaic technologies. Their advantages, including high efficiency, stability, flexibility, and lightweight, offer exceptional prospects for industrial applications [10,11,12]. Among various research areas of CIGSe-based solar cells, completely buffer-free CIGSe cells represent a new concept with limited exploration but significant development potential as a highly simplified device structure [13,14]. This configuration not only streamlines the fabrication process and reduces production costs but also holds promise for enhancing device performance by mitigating optical absorption losses induced by buffer layers [15]. Furthermore, such a simplified device structure facilitates a clearer understanding of the underlying physical mechanisms within CIGSe devices [16].

However, to date, studies on buffer-free solar cells remain scarce, and their efficiencies still lag behind those of classical buffered devices. The primary drawback of buffer-free cells lies in severe recombination at the *p*–*n* junction, necessitating optimization of either the transparent electrode or the absorber [17]. Among the limited reported works, modifying the band alignment at the *p*–*n* junction by doping Mg or S into the conventional ZnO:Al (AZO) transparent electrode has yielded cell efficiencies of 7.96% and 11.3%, respectively [18,19]. As for absorber modifications, Si-doped CIGSe achieved an efficiency exceeding 18% [20]. Si doping forms a grain boundary layer composed of Si-related compounds within CIGSe absorbers, which likely passivates the grain boundaries through the formation of a localized electric field [16]. Additionally, the authors inferred that Si doping may form Si_Cu_ or Si_III_ donor defects and modify the band structure of CIGSe, thus passivating the interface recombination centers.

The enhancement of CIGSe performance via Si doping has broadened the research scope for doping elements in CIGSe absorbers. It is widely acknowledged that elements belonging to the same group generally have similar valence electron structures, thereby usually exhibiting similar chemical properties. Both Si and Ge belong to the IVA group, and extensive reports indicate that Ge and Si exhibit comparable properties in modulating the properties of polycrystalline Cu_2_O thin films [21,22]. Therefore, investigating the impact of Ge doping on CIGSe thin films represents an innovative and worthwhile research direction.

Notably, despite the absence of reported investigations on the impact of Ge doping in CIGSe solar cells, research on Ge-doped CZTSSe devices has been extensively studied because Ge belongs to the same group as Sn [23]. It has been shown that Ge facilitates grain growth by forming a low-melting-point Ge-Se phase [24,25]. Therefore, it is reasonable to speculate that Ge doping in CIGSe precursors may also lead to the formation of a Ge-Se liquid phase during subsequent selenization annealing, which holds the potential to enhance the crystallinity of CIGSe absorbers. Furthermore, the possible Ge-doping-induced point defects in Ge-doped CIGSe absorbers, which may be similar to those observed in Si-doped CIGSe, and their impacts on the band structure and device performance deserve systematic investigation.

In this work, Ge-doped CIGSe absorbers were prepared by stacked sputtering of Ge and CIGSe precursors followed by selenization annealing. Firstly, the influence of Ge doping on the morphology of the CIGSe absorber was investigated, and the phase composition as well as the chemical composition were characterized. Subsequently, to analyze the effect of Ge doping on device performance, completely buffer-free CIGSe solar cells with varying Ge-doping concentrations were fabricated. Such a simplified device structure enabled more straightforward analysis of the impacts induced by Ge doping. The photovoltaic performance and carrier recombination mechanisms of the Ge-doped devices were systematically analyzed.

## 2. Materials and Methods

### 2.1. Fabrication of CIGSe Absorbers and Buffer-Free Solar Cells

CIGSe precursors were fabricated by the magnetron sputtering method on Mo-coated soda–lime glass (SLG) substrates using a Beijing Technol sputtering system equipped with a Huettinger generator. Ge-doped CIGSe precursors were deposited using a stacked sputtering approach. Ge films were first sputtered using a Ge target, followed by the deposition of the CIGSe precursors using a CuInGaSe quaternary ceramic target with the elemental composition of Cu:23.1, In:19.2, Ga:9.0, and Se:48.7 at.%. A schematic diagram of the precursor structure is presented in Figure S1. The Ge-doping concentration was controlled by adjusting the deposition time for Ge films while maintaining a constant deposition time for CIGSe films. Three designed Ge-doping concentrations were 0.23, 0.46, and 0.69 at% (as measured and calculated by ICP-OES), referred to as Ge1, Ge2, and Ge3, respectively. Subsequently, both undoped and Ge-doped CIGSe precursors were annealed in a H_2_Se/Ar atmosphere. The selenization process was conducted at 575 °C with a H_2_Se concentration of 6 vol%. The sputtered ZnO:Al (AZO) films (~500 nm) as the transparent electrode were deposited on absorbers at 200 °C to fabricate buffer-free solar cells. No metal grid or antireflection coating was employed for devices in the work.

### 2.2. Characterization

The morphologies of CIGSe absorbers were examined using scanning electron microscopy (SEM, ZEISS Geminisem 500, Oberkochen, Germany). The measurement was performed under an accelerating voltage of 15 kV and a working distance of approximately 6 mm. The elemental composition was quantified by inductively coupled plasma–optical emission spectrometer (ICP-OES, IRIS Intrepid II, Waltham, MA, USA) and electron probe microanalysis (EPMA, JEOL JXA8230, Tokyo, Japan). Crystalline structures were analyzed by X-ray diffraction (XRD, D8 Advance, Billerica, MA, USA). The composition and chemical states of elements in samples were investigated using X-ray photoelectron spectroscopy (XPS, ESCALAB 250Xi, Waltham, MA, USA), with the spectra calibrated by the standard C_1s_ peak (284.8 eV). The depth distribution of elements in devices was measured by time-of-flight secondary ion mass spectrometry (TOF-SIMS, S9000X, Brno, Czech Republic). The surface morphology and contact potential of absorbers were characterized by atomic force microscopy (AFM, PeakForce Tapping Mode) and Kelvin probe force microscopy (KPFM, Bruker Dimension Icon with Scan Assist, Billerica, MA, USA). Current density–voltage (*J*–*V*) curves were measured under AM 1.5G illumination using a source meter (Keithley 2400, Solon, OH, USA) and a solar simulator (OAI SLPSS-156, Milpitas, CA, USA). Temperature-dependent open-circuit voltage (*V*_OC_–*T*) measurements were performed from 298 K to 138 K using a liquid nitrogen cooling system. Light intensity-dependent *V*_OC_ (*V*_OC_–*G*) measurements were performed using a series of optical transmittance filters (90% to 1%). Capacitance–voltage (*C*–*V*) measurements were conducted in the dark using a source meter (Keithley 4200, Solon, OH, USA). Photoluminescence spectroscopy (PL, HORIBA LabRAM HR Evolution, Kyoto, Japan) was performed at 25 °C with a 785 nm high-power single-frequency OEM laser excitation source. The output power of the laser was 300 mW with the used intensity set to 5%, and the resolution was around 2.1 cm^−1^.

## 3. Results and Discussion

### 3.1. Morphologies and Composition Analysis of Absorbers

**Figure 1:**
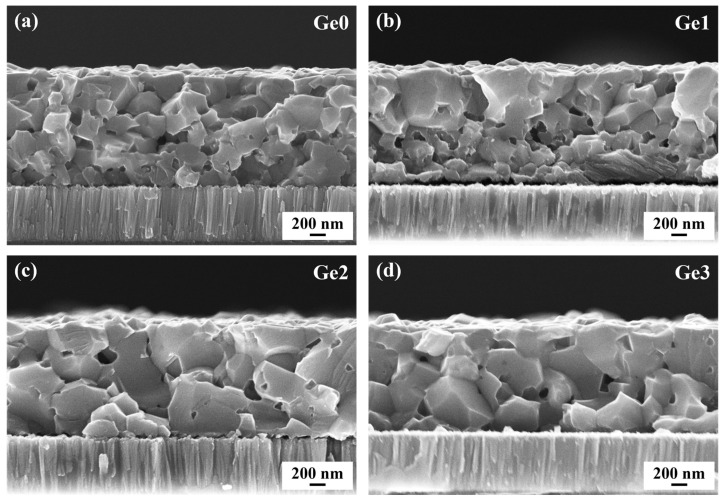
Cross-sectional morphologies of the CIGSe absorbers with different Ge-doping concentrations: (**a**) Ge0, (**b**) Ge1, (**c**) Ge2, (**d**) Ge3.

A notable drawback of CIGSe absorbers prepared via the selenization annealing of CIGSe quaternary ceramic precursors is their relatively poor crystallinity, which significantly limits the enhancement of device performance [26,27]. Figure 1 presents cross-sectional SEM images of undoped and Ge-doped CIGSe absorbers to evaluate the impact of Ge incorporation on morphology. The results demonstrate an obvious improvement in crystallinity upon Ge doping. The undoped sample (Ge0) exhibits a small grain size. With a small amount of Ge doping (Ge1), the grains in the near-surface region become larger, while those in the lower region remain small. As the Ge-doping concentration increases, grain growth extends to the middle and bottom regions. The crystallinity of Ge2 and Ge3 samples is significantly enhanced compared to that of Ge0. These findings indicate that Ge doping at the bottom of CIGSe precursors promotes grain growth in the absorbers, which may be attributed to the Ge-related reactions during the selenization annealing process.

In the research of CIGSe absorbers, it is commonly believed that the formation of a low-melting-point liquid phase during selenization annealing facilitates the diffusion of atoms, thereby assisting grain growth. Studies on Ge-doped CZTSe have demonstrated that Ge and Se form a Ge-Se liquid phase above ~380 °C, acting as a crystallization flux to enhance grain enlargement [23]. Therefore, it can be inferred that Ge in CIGSe precursors might similarly form a Ge-Se liquid phase during selenization annealing, accelerating atomic diffusion in CIGSe films and consequently promoting grain growth. This can explain the larger grains observed in Ge-doped CIGSe absorbers in Figure 1. Such a mechanism is similar to the promoting effects reported for other low-melting-point phases, such as Ag-In-Se and Cu-Bi-Se phases [28,29].

**Figure 2:**
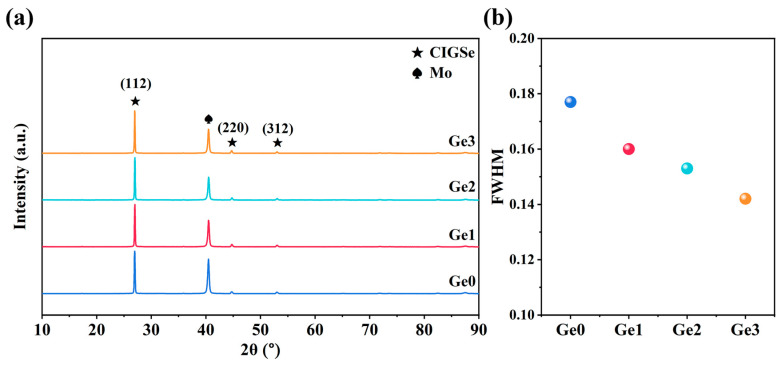
(**a**) XRD patterns and (**b**) FWHM of the (112) peak of samples with different Ge-doping concentrations.

To further investigate the influence of Ge on the phase composition of CIGSe absorbers, XRD measurements were conducted. As shown in Figure 2a, Ge-doped samples exhibit no additional diffraction peaks and no significant shifts in the positions of CIGSe peaks. This indicates that such a low concentration of Ge doping neither introduces impurity phases nor affects the CIGSe lattice structure. Furthermore, Figure 2b reveals that the full width at half maximum (FWHM) of the main CIGSe peak decreases with increasing Ge-doping concentration, which is consistent with the enhanced crystallinity observed in SEM.

**Figure 3:**
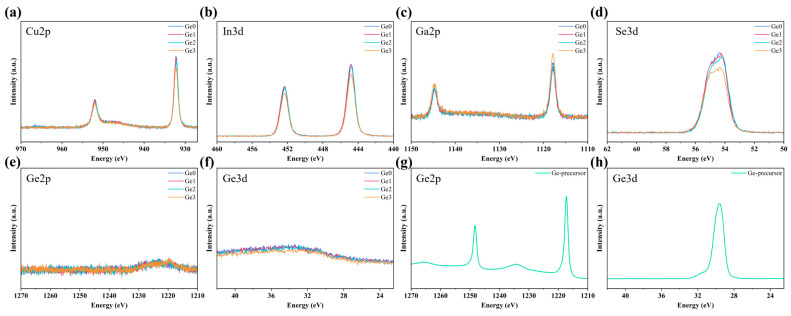
XPS spectra of (**a**–**f**) CIGSe absorbers with different Ge-doping concentrations and (**g**,**h**) Ge precursors.

XPS measurements were conducted to investigate the effect of Ge introduction on the chemical states of elements in CIGSe films, as shown in Figure 3a–f. For comparative evaluation, the XPS spectra of Ge precursors are shown in Figure 3g,h to identify the corresponding binding energy peaks of Ge. The results reveal that Ge doping induces no obvious shifts in the peak positions of Cu, In, Ga, and Se, indicating that the chemical states of these primary elements remain unaffected by Ge doping. Interestingly, no Ge signal was detected in the annealed Ge-doped CIGSe absorbers. Given that XPS is a surface technique, additional EPMA and SIMS measurements were performed to analyze the elemental concentration in absorbers as well as the elemental distribution along the depth direction in devices (Table S1, Figure S2). The results demonstrate that the residual Ge content in the Ge-doped absorber is below the detection limit, suggesting a high proportion of Ge loss during the selenization annealing process. It is suggested that the reaction between Ge and Se does not simply form a Ge-Se liquid phase but also generates a volatile phase (e.g., GeSe or GeSe_2_) at a sufficiently high temperature, which may escape from the absorber [25,30,31]. It is worth noting that the migration of the volatile phase through CIGSe films during the annealing process may also promote grain growth. Such a phenomenon has been reported in the research of Sb-doped CIGSe, which suggests that Sb_2_Se_3_ first transforms into volatile phases and then facilitates grain growth by migrating through CIGSe films [32]. It is noteworthy that the formation and evaporation of volatile phases during annealing may also weaken interfacial adhesion and induce film delamination [33]. As observed from the small gaps at the CIGSe/Mo interface in Figure 1, these non-ideal contact regions could enhance non-radiative recombination and degrade device performance [34]. While Ge-induced volatile phases can facilitate grain growth, they may also introduce adverse effects such as gaps and voids. Therefore, the Ge-doping concentration should be optimized within an appropriate range to mitigate the adverse effects of excessive volatilization.

**Figure 4:**
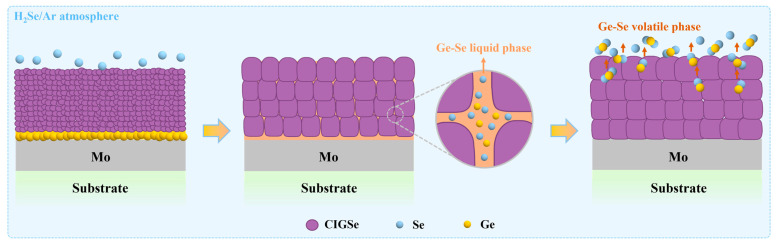
Schematic diagram of the Ge-assisted CIGSe grain growth process during selenization annealing.

Based on these findings, a possible Ge-assisted CIGSe grain growth mechanism is proposed, as schematically illustrated in Figure 4. Under the selenization annealing conditions used in this work, Ge in the precursor reacts with the active Se atoms and forms Ge-Se compounds, which subsequently transform into a Ge-Se liquid phase and volatile phases [25]. It is suggested that the volatile phase typically migrates along grain boundaries. During this process, atoms at the boundaries are perturbed and acquire additional energy, which subsequently leads to more active migration and merging of grain boundaries, resulting in grain size enlargement. Consequently, the formation of both the Ge-Se liquid phase and the volatile phase contributes to the enhanced crystallinity and ultimately results in Ge residue in the annealed absorber that is too low to be detected. This provides a novel approach that effectively promotes CIGSe grain growth without introducing additional impurity phases.

### 3.2. Buffer-Free CIGSe Solar Cells’ Performance

**Figure 5:**
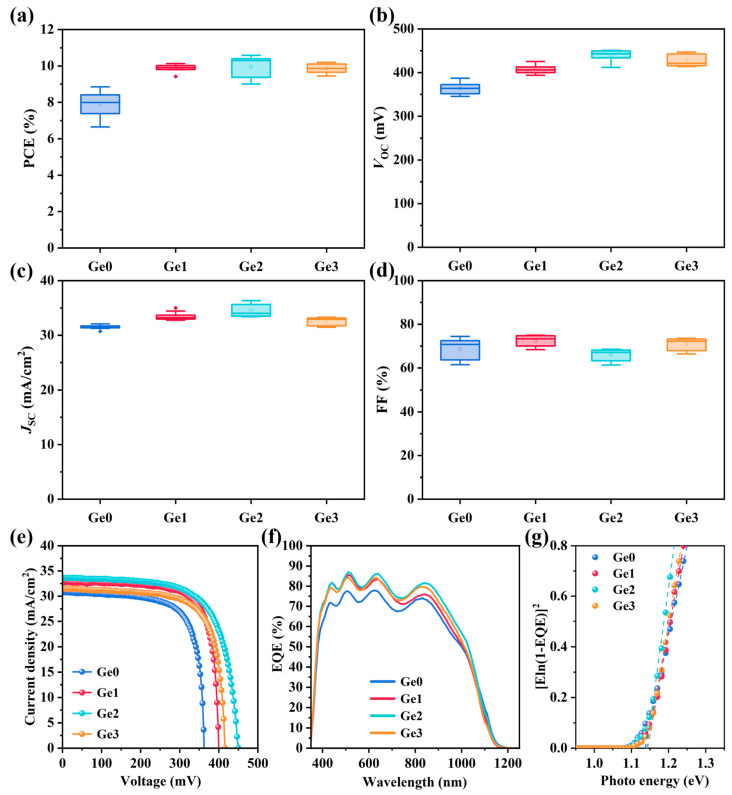
Photovoltaic performance parameters of devices with different Ge-doping concentrations: (**a**) PCE, (**b**) *V*_OC_, (**c**) *J*_SC_, and (**d**) FF. (**e**) *J*–*V* characteristic curves, (**f**) EQE spectrum, (**g**) bandgap derived from the EQE (The dashed lines are linear fitting lines).

Improved crystallinity of CIGSe absorbers is generally recognized to be beneficial for device performance. To study the Ge-doping effects on CIGSe solar cells, buffer-free devices were fabricated using Ge-doped absorbers, and their *J*–*V* characteristics were evaluated. The photovoltaic performance parameters are presented in Figure 5. The results demonstrate a significant enhancement in device efficiency for Ge-doped samples compared to their undoped counterparts, primarily attributed to increases in both *V*_OC_ and *J*_SC_. The Ge2 device achieved the highest efficiency of 10.6%, with an average efficiency increase of 26.2% compared to undoped Ge0 devices. The improvement in *J*_SC_ can be ascribed to the enhanced crystallinity of the absorbers. Grain boundaries usually exhibit relatively complex microstructures and phase compositions, and excessive grain boundaries can scatter carriers, impairing carrier transport and collection efficiency [28]. Consequently, the enlarged grains and reduced grain boundaries resulting from Ge doping contribute to the observed *J*_SC_ improvement. However, as the Ge-doping concentration increases, the changes in *J*_SC_ and FF exhibit a non-monotonic trend. This phenomenon arises because, in addition to enhancing crystallinity, higher Ge-doping concentration also leads to a slight increase in voids in the CIGSe absorber due to the formation of volatile phases, as shown in Figure 1. These voids may increase the device series resistance, which degrades the FF. The competition between these two opposing effects results in the observed non-monotonic trend in FF. In addition, *J*_SC_ is influenced not only by crystallinity but also by carrier recombination within the device. Therefore, the effects of carrier recombination in the device are systematically analyzed and discussed in the following sections.

**Figure 6:**
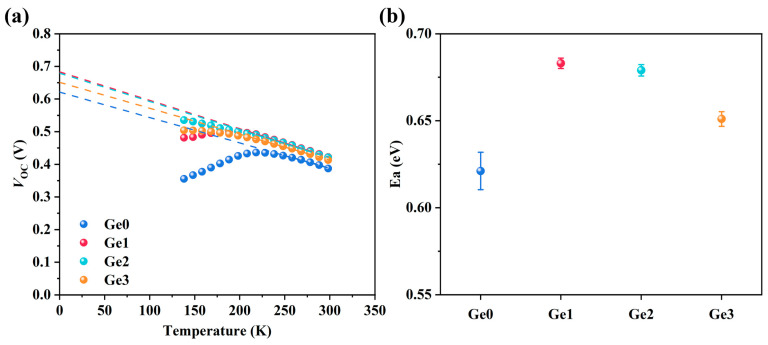
(**a**) *V*_OC_–*T* data of devices with different Ge-doping concentrations and (**b**) the activation energy ($E_{a}$) extracted from *V*_OC_–*T* data.

Additionally, Ge-doped devices exhibit enhanced *V*_OC_. To further elucidate the origin of *V*_OC_ differences and the device recombination mechanisms, *V*_OC_–*T* measurements were conducted. The temperature dependence of *V*_OC_ is described by Equation (1) [35]:

(1) $$V_{OC}=\frac{E_{a}}{q}-\frac{AkT}{q}ln\left(\frac{J_{00}}{J_{L}}\right)$$

where $E_{a}$ is the activation energy, A is the diode ideality factor, $J_{L}$ is the photocurrent density, and $J_{00}$ is the current prefactor. To determine the $E_{a}$ values of devices, *V*_OC_ values at varying temperatures were extracted, as shown in Figure 6a. By linearly fitting the *V*_OC_–*T* data, $E_{a}$ values can be obtained according to Equation (1), as summarized in Figure 6b. The results indicate that the $E_{a}$ values of all devices are lower than the bandgap $E_{g}$ (around 1.14 eV, as measured by EQE (Figure 5g)), suggesting severe interface recombination. This arises from the buffer-free device structure, where the *p*–*n* junction is formed between the CIGSe absorber and the AZO transparent electrode. The band misalignment and lattice mismatch at this heterojunction interface degrade the junction quality, leading to pronounced interface recombination and consequently low $E_{a}$ [17,36]. Nevertheless, it is noteworthy that the $E_{a}$ values vary among samples with different Ge-doping concentrations. Compared to the undoped Ge0 sample, the Ge1 and Ge2 samples show significantly increased $E_{a}$, indicating that low-concentration Ge doping could suppress interface recombination to a certain extent. However, further increasing the doping concentration (Ge3) results in a slightly reduced $E_{a}$, which may imply the introduction of additional defects.

**Figure 7:**
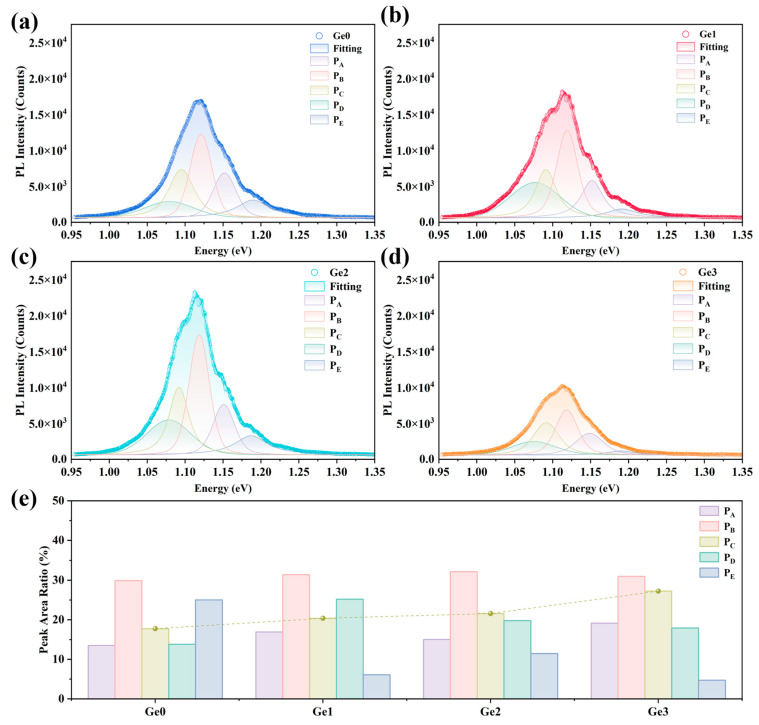
(**a**–**d**) PL spectra of CIGSe devices with different Ge-doping concentrations. (**e**) PL peak area ratio of each sample.

The influence of Ge doping on the band structure and defects of the CIGSe absorber was further analyzed using PL spectroscopy. As shown in Figure 7, peak deconvolution fitting was performed [37], and all spectra exhibit five distinct emission peaks. No obvious additional peaks are detected in Ge-doped samples. Based on their relative spectral positions, the peaks are identified as band-to-band transition (B-B) at 1.15 eV (P_A_), free-to-bound transition related to the acceptor level of Cu vacancy (V_Cu_) at 1.12 eV (P_B_), transition related to the donor level of Se vacancy (V_Se_) at around 1.09 eV (P_C_), and donor-to-acceptor transition (DAP, V_Se_-V_Cu_) at around 1.07 eV (P_D_) [33,38,39]. The low-intensity peak at 1.19 eV is likely attributed to the higher bandgap within the absorber layer, which may result from compositional fluctuations [33,40]. The positions of B-B and V_Cu_ peaks showed no significant shift, which implies that the band structure of CIGSe was not noticeably affected by Ge doping. In addition, Ge doping does not introduce obvious Ge-related defect states, corroborating the earlier observations of Ge loss and negligible Ge remaining within the lattice. However, the peak area ratio of V_Se_ emission is enhanced as Ge-doping concentration increases, which is likely attributed to the Se loss induced by the volatilization of Ge-Se phases during the annealing process. Under identical selenization conditions (H_2_Se concentration and duration), the volatilization of Ge in the form of GeSe or GeSe_2_ compounds concurrently leads to some loss of Se. V_Se_ is a common donor defect in CIGSe absorbers, especially in those fabricated from Se-deficient CIGSe precursors. It is known that the V_Se_ defect level is relatively shallow [41], but an excessively high concentration of V_Se_ may increase carrier recombination in the p-type CIGSe, thereby degrading device performance [42]. This explains the slight decline in both PL intensity and device performance observed in the Ge3 sample. The overall PL intensity of the Ge2 and Ge1 devices increased compared to that of Ge0, indicating a reduction in the device’s non-radiative recombination, leading to an improvement in *V*_OC_ [3]. Although Ge doping does not introduce obvious defects, the associated Se loss is a non-negligible factor affecting photovoltaic performance.

### 3.3. Recombination Analysis of Ge-Doped CIGSe Devices

To further analyze the impact of Ge doping on recombination mechanisms in different regions of devices, the carrier recombination rates were calculated using the model proposed by Grover et al. [43]. Firstly, by equating the total carrier generation to the total recombination at *V*_OC_ bias, Equation (2) is obtained:

(2) $$GW=R^{i}+R^{d}+R^{b}$$

where *G* is the average carrier generation rate; *W* is the absorber thickness; and $R^{i}$, $R^{d}$, and $R^{b}$ are the carrier recombination rates at the interface, the space charge region (SCR), and in the quasi-neutral region (QNR), respectively. Each recombination rate can be further expressed as a product of a bias-independent recombination coefficient and an exponential function of bias *V*, as shown in Equation (3) [44]:

(3) $$\left\{\begin{array}{c} R^{i}=R_{0}^{i}e^{\frac{qV}{kT}}\\ R^{d}=R_{0}^{d}e^{\frac{qV}{2kT}}\\ R^{b}=R_{0}^{b}e^{\frac{qV}{kT}} \end{array}\right.$$

where $R_{0}^{i}$, $R_{0}^{d}$, $R_{0}^{b}$ are the carrier recombination rate coefficients at the interface, in the SCR, and in the QNR, respectively. These parameters could represent the intrinsic characteristics of the recombination behavior in devices. *T* is the measurement temperature. By combining Equations (2) and (3) at *V*_OC_ bias, *V*_OC_ can be expressed as Equation (4) [45]:

(4) $$V_{OC}=\frac{2kT}{q}ln\left[\frac{1}{2}\frac{R_{0}^{d}}{R_{0}^{i}+R_{0}^{b}}\left(\sqrt{4GW\frac{R_{0}^{i}+R_{0}^{b}}{{\left(R_{0}^{d}\right)}^{2}}+1}-1\right)\right]$$

**Figure 8:**
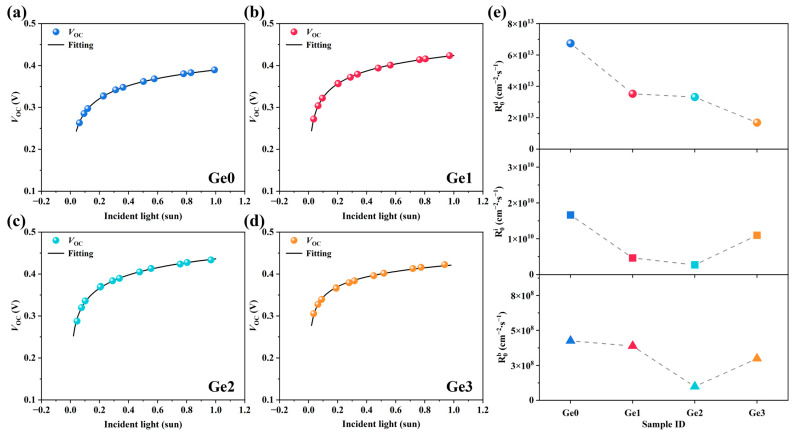
(**a**–**d**) *G*–*V*_OC_ data and fitting curves of CIGSe devices with different Ge-doping concentrations and (**e**) the variation trend of recombination rate coefficients of devices.

*J*–*V* curves of devices with different Ge-doping concentrations were measured under varying light intensity *G*. The extracted *V*_OC_ values corresponding to different *G* are presented in Figure 8a–d. By fitting Equation (4), $R_{0}^{d}$, and ($R_{0}^{i}{\;+\;R}_{0}^{b})\;$ can be derived. The fitted curves match the functional model well, and the root mean square error (RMSE) was no greater than 0.002. However, $R_{0}^{i}$ and $R_{0}^{b}$ cannot be distinguished based on Equation (4). We use Equation (5) to further calculate $R_{0}^{i}$ and $R_{0}^{b}$ [44].

(5) $$E_{a}=\frac{R_{0}^{i}q\varphi_{b0}+R_{0}^{b}E_{g}}{R_{0}^{i}+R_{0}^{b}}$$

where $E_{a}$ can be obtained from the *V*_OC_–*T* data shown in Figure 6, $E_{g}$ is the bandgap determined from the EQE (Figure 5g), and $\varphi_{b0}$ is the hole barrier at the *p*–*n* junction interface, which can be calculated from Equation (6) [46]:

(6) $$\varphi_{b0}=\varphi_{bi}+\frac{E_{F}+E_{V}}{q}=\varphi_{bi}+\frac{kT}{q}ln\frac{N_{V}}{N_{A}}$$

where $\varphi_{bi}$ is the built-in voltage, $N_{V}$ is the effective density of states in the valence band of CIGSe, and $N_{A}$ is the majority carrier density in the absorber. Both $\varphi_{bi}$ and $N_{A}$ can be derived from the linear fitting of 1/*C*^2^–*V* curves through *C*–*V* measurements (as shown in Figure S3).

By combining Equations (4) and (5), $R_{0}^{d}$, $R_{0}^{i}$, and $R_{0}^{b}$ for each device were calculated and summarized in Figure 8e. The introduction of Ge leads to a reduction in recombination rate coefficients to varying degrees, indicating an overall improvement in device quality with Ge doping. As the Ge-doping concentration increases, $R_{0}^{d}$ decreases significantly. This reduction is attributed to the enlarged grain size in Ge-doped CIGSe absorbers, particularly the improved crystallinity in the near-surface region. The reduction in recombination occurring at grain boundaries substantially suppresses the recombination in the SCR. However, $R_{0}^{i}$ and $R_{0}^{b}$ exhibit a non-monotonic trend with increasing Ge-doping concentration.

**Figure 9:**
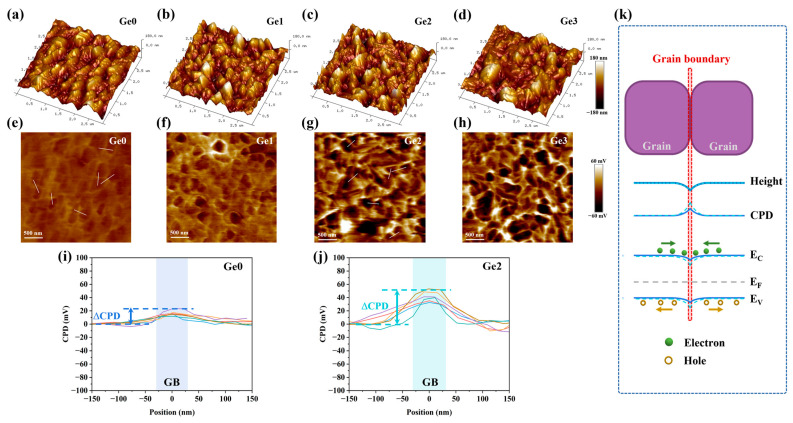
(**a**–**d**) AFM and (**e**–**h**) KPFM images of samples with different Ge-doping concentrations; (**i**,**j**) CPD line profiles (the white lines) of Ge0 and Ge2 samples (aligned to a common base value, each colour represents a different extracted line); (**k**) the schematic diagram of the energy band structure of the grain boundary.

AFM and KPFM measurements were performed to further investigate the mechanisms of Ge-doping effects on the recombination rate. Figure 9a–h show the surface height map and the contact potential difference (CPD) distribution of the undoped sample (Ge0) and the Ge-doped samples (Ge1, Ge2, and Ge3). The CPD at grain boundaries (GBs) is higher than that within the grains for all samples. This indicates a downward energy band bending at the GBs [47,48], as illustrated in Figure 9k. The downward band bending results in a local electric field at the GB that creates a hole barrier and attracts electrons [49]. Such an electric field can effectively suppress electron–hole recombination at GBs, thereby promoting more efficient carrier separation and transport. As shown in Figure 9e–h, the CPD between the GB and the grain interior (ΔCPD) progressively increases with the increasing Ge-doping concentration. A larger ΔCPD corresponds to a stronger local electric field, which is more favorable for suppressing recombination [48]. To present this difference more clearly, representative statistical results of multiple line profiles extracted from the Ge0 and Ge2 samples are shown in Figure 9i and Figure 9j, respectively. It is demonstrated that ΔCPDs of Ge2 are notably higher than their Ge0 counterparts. This suggests that Ge doping induces stronger band bending at the GBs of CIGSe absorbers, which further reduces carrier recombination.

Downward band bending at GBs is generally attributed to the existence of positively charged cations or donor defects [50]. For example, the band bending at CIGSe GBs has been attributed to Na_i_^+^ interstitial defects [51,52], whereas in Ge-doped CZTSe, a similar effect is considered to be caused by Ge^4+^ cations [53]. Ge^4+^ cations in CIGSe may exert similar effects. However, given the retention of Ge in the absorber is too low to be detected in this study, the enhanced band bending at GBs is more likely attributed to the V_Se_^2+^ donor defects, which may have similar effects to Na_i_^+^ [47]. As indicated by PL measurements, the V_Se_ defects are increased in Ge-doped CIGSe. Therefore, we could deduce that the diffusion and volatilization of Ge-Se compounds along GBs leave behind more V_Se_ defects at these sites.

The combination effects of improved crystallinity and grain boundary passivation induced by Ge doping contribute to the suppression of recombination and the enhancement of device performance. Given that the absorber surface typically contains more GBs, the reduction in GB density and passivation of GBs also lowers $R_{0}^{i}$ in Ge-doped devices. For Ge1, crystallinity is improved in the near-surface region, while the middle and bottom regions still consist of small grains. As a result, $R_{0}^{d}$ decreases, whereas the change in $R_{0}^{b}$ is relatively less pronounced. For Ge2, the crystallinity in the whole absorber is improved, leading to a more notable reduction in both $R_{0}^{d}$ and $R_{0}^{b}$, which corresponds to the highest device performance in this work. However, Ge3 exhibits relatively increased recombination rates due to the adverse effects of excessive V_Se_ defects, leading to a slight decrease in both *V*_OC_ and *J*_SC_, and ultimately resulting in device efficiency degradation.

The results suggest that further improvement should focus on optimizing the selenization annealing conditions for samples with higher Ge-doping concentration to prevent excessive formation of V_Se_ defects, thereby better exploiting the beneficial effects of Ge doping.

## 4. Conclusions

In summary, this work presents an effective approach for grain growth in CIGSe thin films through Ge doping. Compared to undoped samples, Ge-doped CIGSe precursors exhibit significantly improved crystallinity after selenization annealing. This enhancement is likely attributed to the formation of the Ge-Se liquid phase and volatile phase during selenization, which promotes grain growth. Moreover, owing to the low Ge-doping concentration and the volatility of Ge-Se compounds, Ge residue is undetectable in Ge-doped absorbers. Thus, Ge doping offers a method to improve crystallinity without introducing notable impurity phases or Ge-related defects. However, Ge loss through volatilization of Ge-Se compounds during selenization also induces Se loss and increased Se vacancy defects. An excessive concentration of such donor defects may degrade the performance of the p-type CIGSe absorber and increase carrier recombination, which may degrade the device performance. In addition, higher contact potential differences at grain boundaries in the Ge-doped absorber are identified, which is considered beneficial for reducing carrier recombination at grain boundaries. The combination effects of enhanced crystallinity and passivated grain boundaries lead to a marked reduction in the carrier recombination rates of Ge-doped buffer-free CIGSe devices, leading to enhanced device performance. Nevertheless, further increasing Ge-doping concentration accentuates the adverse effects of Se loss, causing a slight degradation in device efficiency. Ultimately, based on the beneficial effects of Ge doping, the efficiency of buffer-free CIGSe solar cells has increased to 10.6%, with an average efficiency improvement of 26.2% compared to undoped devices. Future research should focus on optimizing the selenization annealing process for higher Ge-doping concentrations to ensure sufficient Se supply, thereby suppressing the formation of excess Se vacancies. This approach would allow the beneficial effects of Ge doping to be fully realized, including promoting grain growth and suppressing recombination, while minimizing its adverse impacts, ultimately maximizing the positive contribution of Ge doping to device performance.

## Data Availability

The original contributions presented in this study are included in the article/Supplementary Materials. Further inquiries can be directed to the corresponding authors.

## Supplementary Materials

The following supporting information can be downloaded at: https://www.mdpi.com/article/10.3390/ma19030499/s1. Figure S1: The schematic diagram of the Ge-doped precursor structure and the fabrication process of CIGSe buffer-free devices.; Figure S2: (a-d) SIMS depth profiles of devices with different Ge concentrations; (e) the extracted Ge profiles.; Figure S3: 1/*C*^2^ versus bias voltage profiles derived from the *C*-*V* data of devices with different Ge-doping concentrations; Table S1: Elemental concentration in CIGSe absorbers with different Ge-doping concentrations measured by EPMA.
