# Supplementary material for: A Novel Ge-Doping Approach for Grain Growth and Recombination Suppression in Buffer-Free CIGSe Solar Cells
Source: Materials (Basel). 2026 Jan 27;19(3):499. doi: 10.3390/ma19030499 (PMC12898081; doi:10.3390/ma19030499)
Supplement: Supplementary file 1 [file materials-19-00499-s001.zip › materials-4097617-supplementary.pdf]

Supplementary Materials

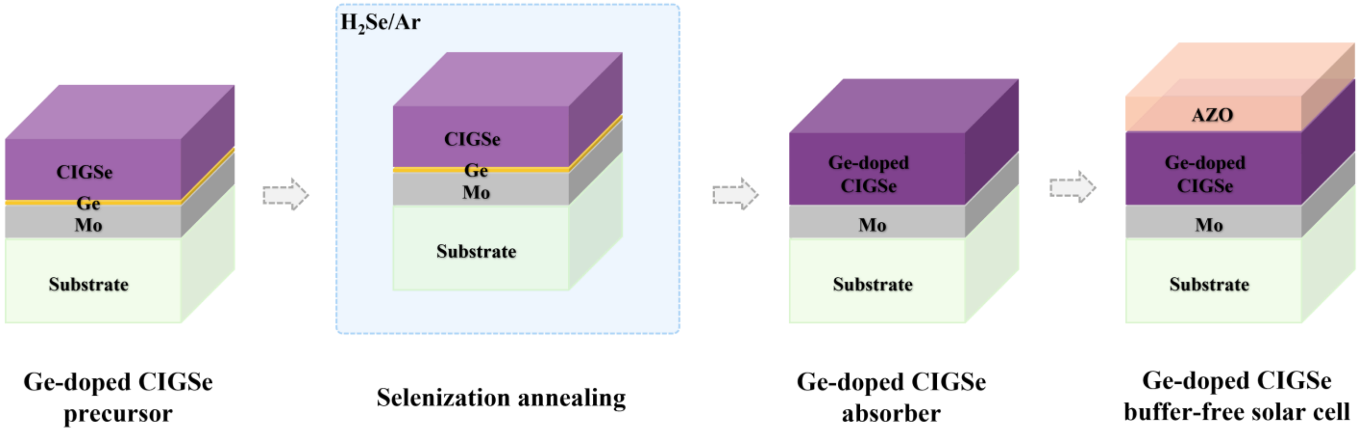

Supplementary Figure S1. The schematic diagram of the Ge-doped precursor structure and the fabrication process of CIGSe buffer-free devices.

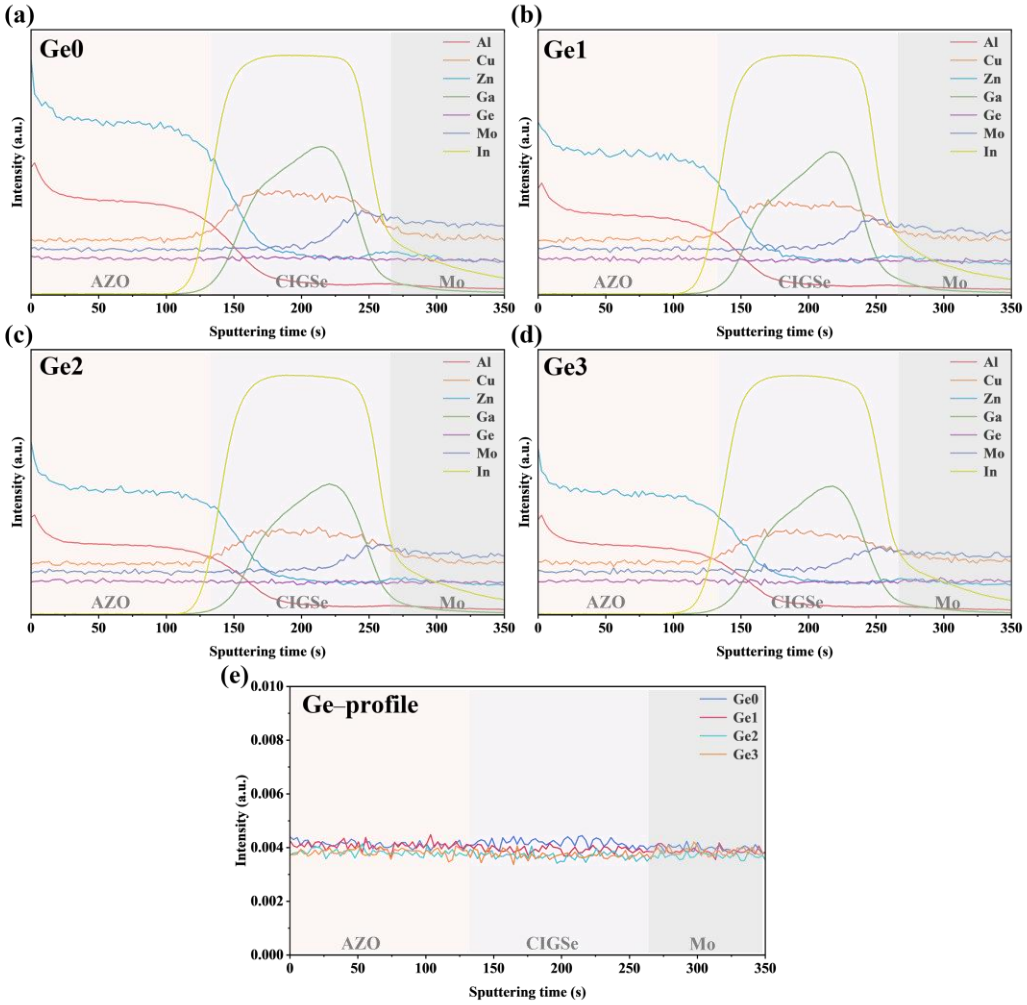

Supplementary Figure S2. (a-d) SIMS depth profiles of devices with different Ge concentrations; **(e)** the extracted Ge profiles.

Supplementary Table S1: Elemental concentration in CIGSe absorbers with different Ge-doping concentrations measured by EPMA.

Sample ID	Cu (at%)	In (at%)	Ga (at%)	Se (at%)	Ge (at%)
Ge0	23.742	20.214	6.488	49.544	$<$0.015
Ge1	24.116	20.806	5.993	49.078	$<$0.015
Ge2	24.266	20.606	6.075	49.043	$<$0.015
Ge3	23.990	20.632	6.286	49.082	$<$0.015

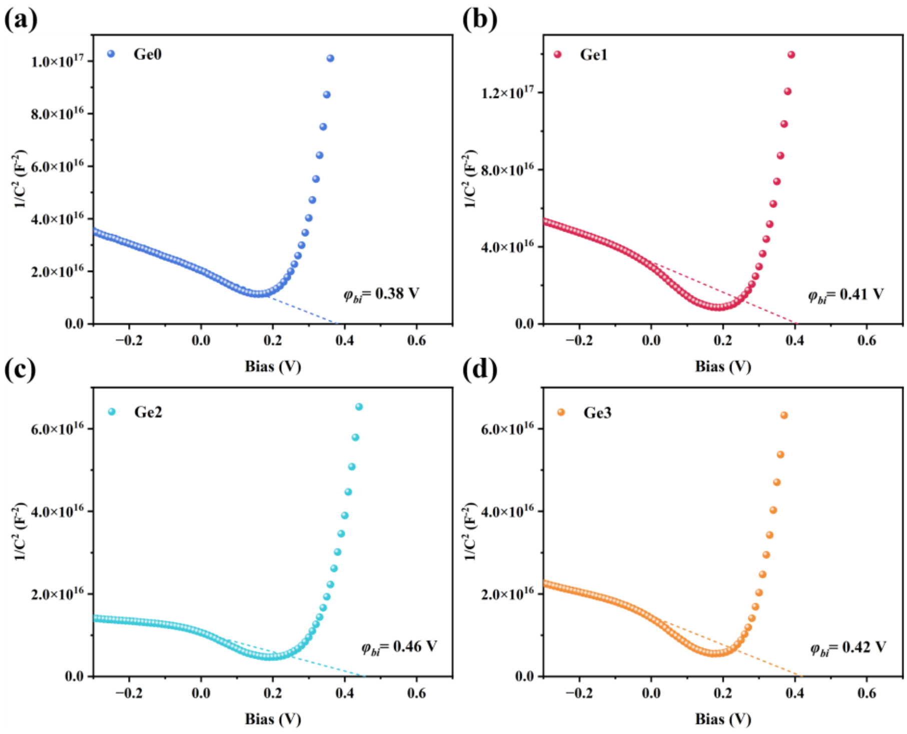

Supplementary Figure S3. 1/C^2 versus bias voltage profiles derived from the *C*-*V* data of devices with different Ge-doping concentrations.
